# The yin and yang of B cells in a constant state of battle: intestinal inflammation and inflammatory bowel disease

**DOI:** 10.3389/fimmu.2023.1260266

**Published:** 2023-10-02

**Authors:** Roxana Zogorean, Stefan Wirtz

**Affiliations:** ^1^ Medizinische Klinik 1, Universitätsklinikum Erlangen, Friedrich-Alexander-Universität Erlangen-Nürnberg, Erlangen, Germany; ^2^ Medical Immunology Campus Erlangen, FAU Erlangen-Nürnberg, Erlangen, Bavaria, Germany

**Keywords:** regulatory B cells (Bregs), IgA, microbiota, IBD, intestinal inflammation, IgG, plasma cells

## Abstract

Inflammatory bowel disease (IBD) is a chronic inflammatory disease of the gastrointestinal tract, defined by a clinical relapse-remitting course. Affecting people worldwide, the origin of IBD is still undefined, arising as a consequence of the interaction between genes, environment, and microbiota. Although the root cause is difficult to identify, data clearly indicate that dysbiosis and pathogenic microbial taxa are connected with the establishment and clinical course of IBD. The composition of the microbiota is shaped by plasma cell IgA secretion and binding, while cytokines such as IL10 or IFN-γ are important fine-tuners of the immune response in the gastrointestinal environment. B cells may also influence the course of inflammation by promoting either an anti-inflammatory or a pro-inflammatory milieu. Here, we discuss IgA-producing B regulatory cells as an anti-inflammatory factor in intestinal inflammation. Moreover, we specify the context of IgA and IgG as players that can potentially participate in mucosal inflammation. Finally, we discuss the role of B cells in mouse infection models where IL10, IgA, or IgG contribute to the outcome of the infection.

## Introduction

Inflammatory Bowel diseases (IBD) are chronic inflammatory disorders of the gastrointestinal tract. Several factors may essentially contribute to the onset and progression of IBD: genetic factors, environmental factors, the host immune system and microbiota. The crosstalk between microbiota and the immune cells together with intestinal epithelial cells comprises the hallmark of intestinal homeostasis (1, 2). Although this interaction is essential for maintaining a balanced system and for initiating a protective response to luminal pathogens, it seems to be also critical in the pathogenesis of IBD. In response to intestinal barrier disruption, cytokines and chemokines are released in excess and are crucial contributors to acute intestinal inflammation (3). IBD patients are unable to resolve the inflammation which further develops into chronic intestinal inflammation due to sustained activation of the mucosal immune cells.

Importantly, inflammatory bowel diseases, Crohn’s disease and ulcerative colitis, are characterized as well by increased antibody responses at the mucosal site which participate in exacerbation of inflammation (4–8). Despite the fact that the pathogenic role of T cell subsets and soluble factors such as TNF and IL-23 have been intensively studied in intestinal inflammation (9–11), the role of B cells and antibodies still remains largely elusive. However, in recent years, studies have increasingly brought more light into the role of B cells in IBD. In particular the detection of autoantibodies in the serum of both ulcerative colitis (UC) and Crohn’s disease (CD) patients has led to a better understanding of possible therapeutic strategies (12). Different pathophysiology defines the two types of IBD in regards to autoantibodies: UC patients have higher IgG antibodies against tropomyosin 1 and 5 isoforms (13, 14) and anti-neutrophil cytoplasmic antibody (ANCA) (15). On the other hand, CD patients have much lower ANCA antibodies but increased levels of IgG and IgA against Saccharomyces cervisiae (16), flagellin (17) and E. coli (18). However, more studies are necessary for elucidating the pathogenic role and the relevance of autoantibodies in IBD (12).

Due to the disruption of the intestinal barrier in IBD, increased levels of plasma cells are found in the mucosa of UC patients and may be an indicator of severity or relapse (8, 19). In addition to the disruption of the intestinal barrier, IBD patients have altered microbiota composition compared to healthy controls (20–22). IgA, the most abundant antibody in the body, has been shown to have a role in targeting bacterial species to maintain homeostasis and control and elimination of pathogenic bacteria (23). The fitness of beneficial bacteria is an important parameter for health, and the secretion of IgA may have an advantageous role to play in this respect. While several commensal bacteria are known to play an immunosuppressive role in inflammation and the imbalance between beneficial and harmful bacteria is a feature of IBD, the precise function of gut bacteria in IBD still remains incompletely understood (24).

In homeostasis, B cells are involved in maintaining the intestinal barrier by producing antibodies such as IgA and IgM. In addition, they have immunosuppressive activity as regulatory B cells (Bregs) during inflammation, and their role has been described in a number of diseases, including IBD, in both humans and mice (25, 26). B cells have been suggested to be involved in the immunoregulatory response to intestinal inflammation through the production of the anti-inflammatory cytokine IL-10 in T cell-induced colitis or DSS colitis models (25, 26). B-cell depletion using anti-CD20 therapies also targets Bregs and IL-10, leading to increased CD4+ T-cell proliferation and proinflammatory cytokines and worsening UC (27, 28). In comparison to regulatory T cells, Bregs do not consist of a single subset of B cells (29) which raises the interest for their possible hindered functions.

However, in a murine model of IBD, B cells are found to be increased in the mesenteric lymph nodes and may increase the inflammation severity together with T cells (30). A recent study found that B cells with an IFN-I signature reduced the stromal-epithelial cell interaction required for mucosal healing. The study demonstrates the short-term beneficial effect of B cell depletion on wound healing in inflamed colonic tissue (31).

IgA is produced mainly in the gut-associated lymphoid tissues (GALT) such as Peyer’s patches, mesenteric lymph nodes (mLN), isolated lymphoid follicles (ILF), the cecal patch but also in the lamina propria. In mice, the germinal centers formed in the Peyer’s patches and mLN support the differentiation of IgA^+^ B cells and consequently the production of specific IgA, while ILF seem to contribute mostly to T-independent IgA synthesis (32, 33). Interestingly, human GALT ILFs have recently been classified into two subtypes, namely mucosal ILF (M-ILF) and submucosal ILF (SM-ILF). These, ILFs have been shown to contain germinal centers and memory B cells and are a major source of physiologically important IgA (34) indicating the existence of substantial species specific differences.

Although the function antibodies in the intestine environment may be better understood, acting in opsonization of pathogens, recruiting complement, binding Fc receptors (35–38), the function of IgA is still puzzling especially due to the dichotomic role of maintaining tolerance to commensals and elimination of pathogens.

Studies done on activation induced cytidine deaminase-deficient mice showed that IgA is important for modulating the bacterial communities in the intestine. Lack of IgA in the AID ^-^/^-^ mice determined the excessive growing of anaerobic bacteria in the small intestines. Colonization of germ-free mice with the same anaerobic bacteria showed strong induction of unspecific IgA (39). The changes in the microbial environment caused strong activation of the immune response with hypertrophy in the lymphoid tissues.

IgA modulates the microbiota-immune response cross talk by acting on bacterial epitopes and on bacterial metabolism (40, 41). For example IgA directed against the capsular polysaccharide CPS4 determines a less pro-inflammatory phenotype of Bacteroides thetaiotaomicron (42). IgA antibodies protect against colonizing mucosal pathogens and maintain a homeostatic environment for commensals using a variety of strategies that have been reviewed elsewhere (43, 44). An interesting observation was made by Bunker et al. when they tested the binding affinities of IgA antibodies to bacteria *in vitro*. The study shows that the antigen expression levels on bacteria that drives IgA binding are depending on the environment (45).

Herein, we review how pro-inflammatory cytokines may play an anti-inflammatory or pro-inflammatory role in the intestine and how microbiota is influenced by immune cells interaction and in return can influence the nature of immune response. In addition, we discuss regulatory B cells in relation to microbiota. We present newly described IgA producing Bregs as possible key players in shaping the microbiota to enhance suppression of inflammation.

## Anti or pro-inflammatory cytokines: fine-tuners of the immune response

Cytokines and chemokines enable intercellular communication and participate in maintaining homeostasis of the intestine. In IBD patients, their functions seemed to be skewed to a continuous inflammatory state with negative effects on intestinal barrier integrity (3). However, the labels of pro- and anti-inflammatory describe a simplistic view of cytokines functions during an inflammatory process (46). When sensing microbial components through e.g. Toll like receptor (TLR) signaling, lamina propria macrophages and dendritic cells produce large amounts of IL-1β, IL-6, IL-18 and TNF (47). IL-6 is a pro-inflammatory cytokine which can activate antigen presenting cells and T cells from the intestine and may also have a function in the stimulation and expansion of intestinal epithelial cells (IEC) (48). However, blocking of IL-6 or IL-6 signaling has become an important direction especially in treatment of UC and CD patients with high production of IL-6 (49).

TNF is produced mostly by CD14+ macrophages, adipocytes, T cells, fibroblasts in patients with IBD (50–52) and has several effects in inducing intestinal inflammation in colitis: causing necroptosis in Paneth cells, activating macrophages and effector T cells, affecting the IEC and disrupting the intestinal barrier. IBD patients treated with anti-TNF antibodies show reduced inflammation (53), but the treatment with soluble TNF receptor has failed to improve the disease (54). It was thus hypothesized that soluble TNF is needed for self-renewal during intestinal inflammation while anti-TNF antibodies resolve inflammation by binding to membrane TNF on immune cells thereby causing apoptosis (55).

Innate lymphoid cells (ILCs) have been shown to produce high levels of mainly pro-inflammatory cytokines such as IFN-γ and IL23 which drive experimental innate immune mediated colitis (56). IL23 production has been found to be increased in several murine models of colitis, suggesting an essential role in intestinal inflammation (57–59). Furthermore, UC and CD patients have increased levels of IL23 in the serum and mucosa (60, 61). Several other mechanisms were described that render the balance to a pro-inflammatory state: the production of CC-chemokine ligand 3 by ILCs recruits CCR1+ inflammatory monocytes and intensify the inflammation in CD (62).

Moreover, in mice type 3 innate lymphoid cells are one of the primary producers of IL-22, a cytokine with pleiotropic effects mainly on non-hematopoietic cells (63). IL-22 production has protective roles in the context of mucosal inflammation by preserving intestinal stem cells after tissue damage (64) and by producing antimicrobial peptides which act on the intestinal barrier and microbiota (65, 66). Besides its beneficial role, studies however indicate that overexpression of IL-22 results in high antimicrobial peptides production with loss of bacterial diversity in IL10^-^/^-^ mice, which develop spontaneous colitis compared to IL10^-^/^-^IL-22^-^/^-^ mice (67).

Studies have revealed high numbers of T cells present in the intestine of IBD patients together with high levels of T cell-derived pro-inflammatory cytokines (68, 69). Interestingly, there are couples of differences in T cells subtypes present in CD compared to UC patients. Th1 infiltration and increased production of IFN-γ and IL-2 is detected mostly in the lamina propria of patients of CD (70), while NKT cells with Th2 type phenotype seem to be present in the lamina propria of UC patients (71, 72).

Another T cell subset, Th17 cells, has been shown to produce high levels of cytokines with pro-inflammatory activity in mouse models and potentially in both CD and UC (73, 74). Th17 cells, together with other types of immune cells such as γδ T cells and ILCs, are the major producers of IL-17 in response to stimulation by IL-1β and IL-23. During inflammation, IL-17 plays a role in recruiting neutrophils, antimicrobial peptide production and maintaining the intestinal barrier (75). Although IL-17 production protects against bacterial or fungal intestinal infections, it can also have a pathological role. High levels of IL-17 are found in the serum and in the mucosa of patients with UC and CD (61, 76). Whether its supports inflammation in IBD remains however unclear (77).

Integrity of the intestinal barrier is maintained also by γδ T cells, a cell type abundant in the intestinal epithelium. These cells were shown to produce antimicrobial peptides or cytokines such as IL-17, but can also contribute to intestinal immune reactions via molecules such as granzymes (78). Despite their described role in maintaining intestinal homeostasis in mice (79), their distribution in IBD patients is less clear (80). For instance, one study found increased expression of gut-homing chemokine receptor 9 on circulating γδ T cells in IBD patients (81). However, another recent study, using single cell sequencing, showed a decrease of CD8+ T cells and γδ T cells in the tissues of patients with severe CD (82). Nonetheless, treatment of CD patients with a neutralizing anti–IL-17 receptor monoclonal antibody has shown no efficacy and even worsened disease (83). Moreover, murine and human Th17 cells may produce a more diverse pro-inflammatory repertoire of cytokine such as IL-26 and IFN-γ (84–87) suggesting a strong role in inflammation and tissue damage in IBD.

The role of IL-26 in intestinal inflammation may depend on the disease state, with a protective effect in acute inflammation but damaging in the chronic inflammation of IBD patients (88). While IL-8 is known to have a role in immune cell recruitment to the site of infection or inflammation (89), it also has a role in differentiation of Caco-2 BBE monolayers to enterocytes (90). Although, IL-1β levels are elevated in IBD patients and are corelated with disease severity, macrophage derived IL-1β signals from sensing microbiota in steady state promote Treg differentiation and tolerance to dietary antigens (91).

Importantly, non-immune cells contribute to the production of pro-inflammatory cytokines in IBD (92). These cells may activate the immune cells by producing TNF and IL-6 or may respond to pro-inflammatory cytokines produced by lymphocytes or APC and thus participate in intestinal barrier destruction in IBD. On the contrary, IL-6 production by murine intestinal epithelial cells promoted crypt organoid proliferation and increased stem cell numbers (93). Moreover, anti-inflammatory cytokines such as IL-10 are induced in response to microbiota and participate in maintaining tolerance and homeostasis in the murine intestinal epithelium (94, 95).

In conclusion, the cytokine network involved in intestinal inflammation which then leads to IBD is regulated in a complex manner by genetic, microbial and immune factors. Therapeutic agents are used to block cytokines or cytokine signaling but have sometimes limited efficacy or different outcomes depending on patient subgroups (3, 96).

## The role of microbiota in maintaining homeostasis

**Figure 1:**
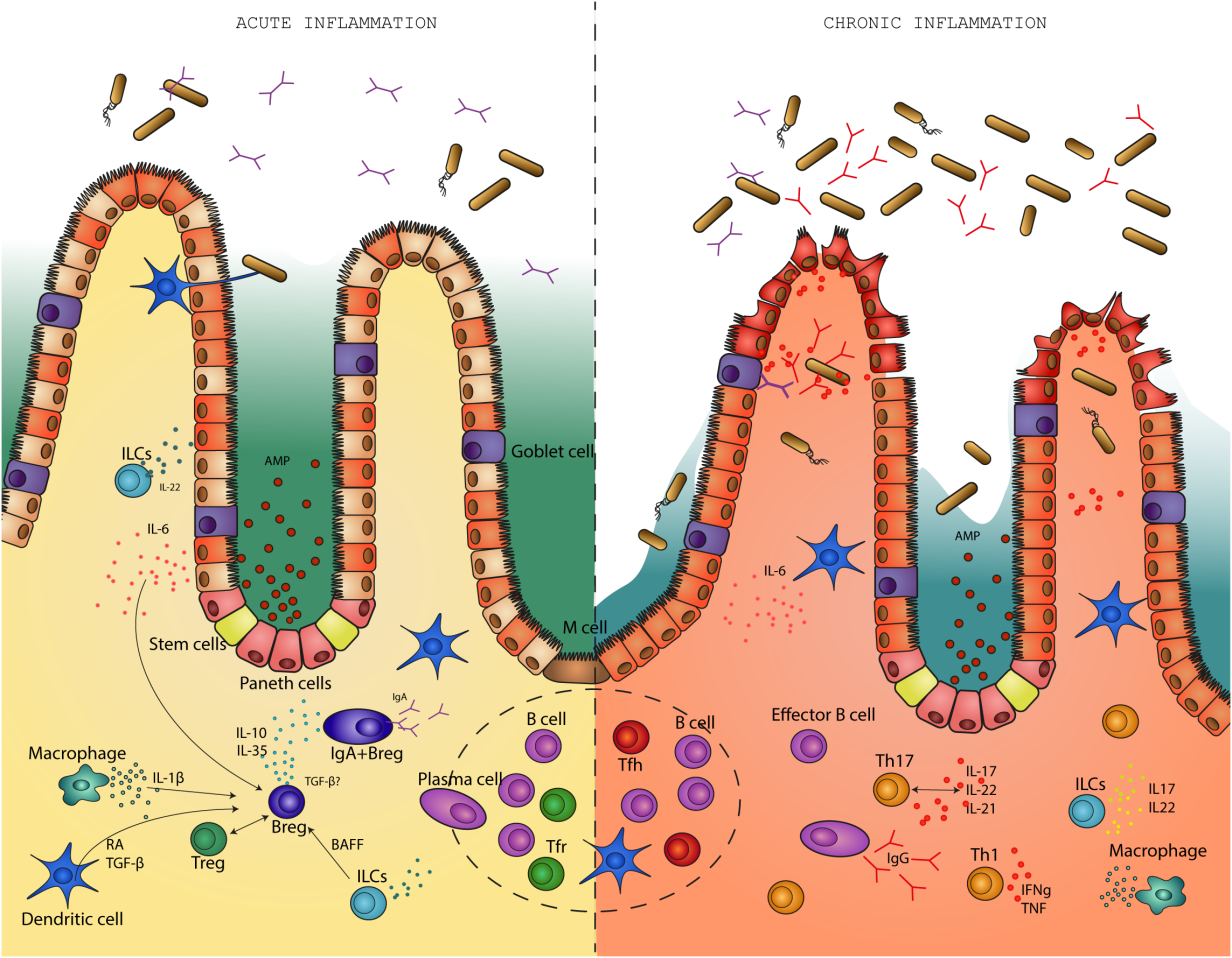
Regulatory B cells differentiate in response to pro-inflammatory stimuli during an acute infection. In a feedback loop with the microbiota, regulatory B cells suppress inflammation in the gut and help return the environment to homeostasis. On the other hand, in the absence of regulatory B cells, intestinal inflammation becomes chronic. This is associated with disruption of the intestinal barrier, bacterial translocation and high levels of pro-inflammatory cytokines. B effector cells produce high levels of IgG antibodies in response to disruption of the mucosal barrier.

Homeostasis in the gut is characterized by immune tolerance to commensal bacteria while preserving an intact intestinal barrier and successfully fighting and clearing out pathogens. The intestinal microbiota is separated from the intestinal epithelium by a barrier of mucus (97, 98) enriched in antimicrobial peptides (AMPs). Not solely the mucus layer acts as shield, but is also the environment where bacteria are constrained by immune response (**Figure 1**).

**Figure 2:**
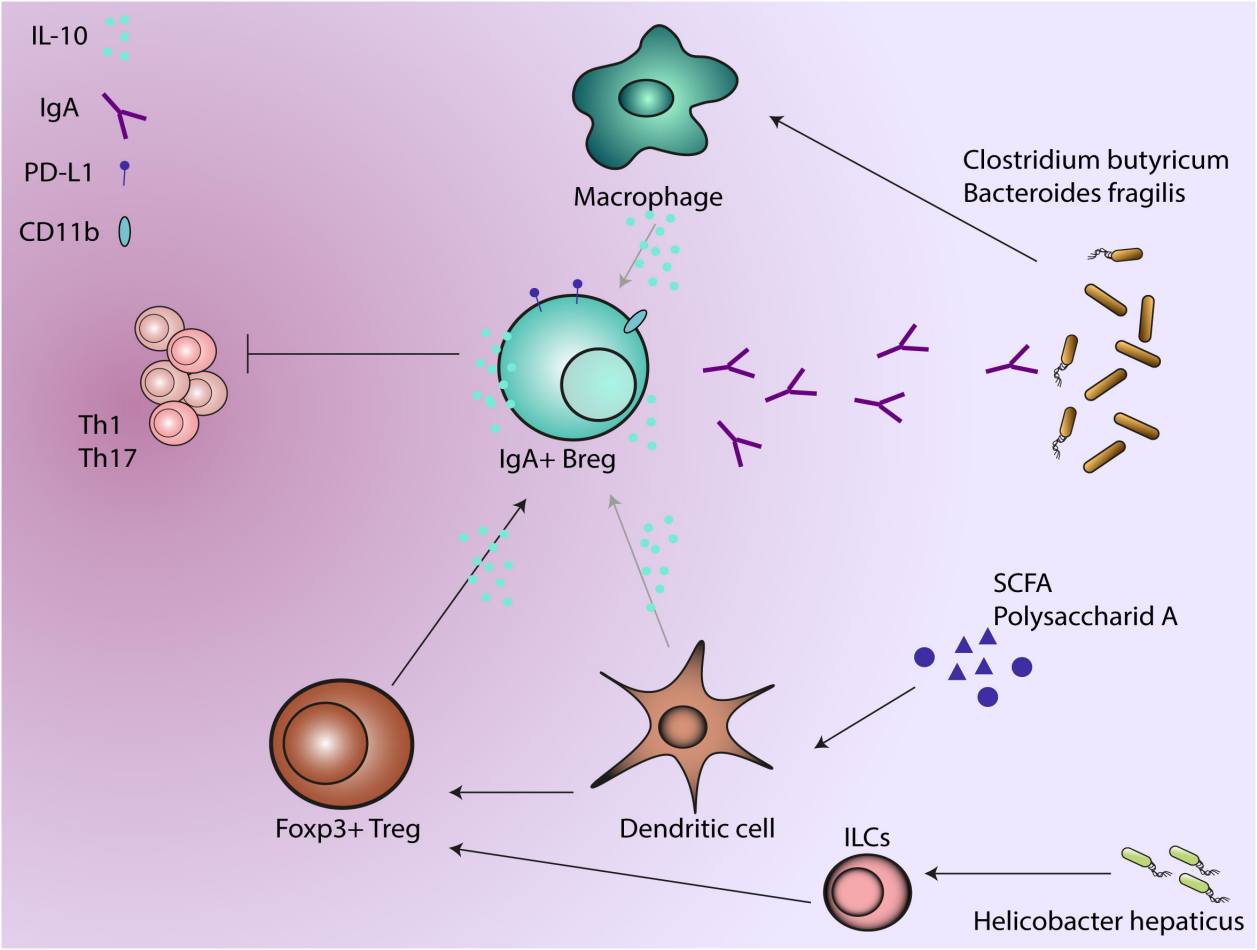
Regulatory B cells produce IgA in response to anti-inflammatory factors such as IL-10. As a dual protective phenotype, IgA production regulate the microbial composition while IL-10 production suppresses the activity of immune cells. Microbiota and microbial metabolites induce regulatory phenotype in other immune cells that further support IgA+ B regs, which will further regulate the microbiota and suppress the activity of T cells.

Various intestinal dendritic cell (DC) subsets capture luminal antigens from commensal bacteria via several proposed mechanisms. One pathway refers to the delivery of luminal antigens and is dependent on M cells, neonatal Fc receptors or apoptosis. It was demonstrated recently that DC sampling of both soluble and particulate antigens via so called Goblet cell associated Passages (GAPs) could be of particular importance for mucosal immunity and tolerance (99, 100). The direct pathway involves intestinal dendrite extension into the lumen to sample antigens (101) followed by migration to the lymph nodes (102). Importantly, intestinal DC functions support IgA production by B cells (103). Commensal products such as polysaccharide A condition DCs to induce IL-10 producing T regs which help in preventing colonic inflammation in the mouse colitis models (104–106). Murine DCs can be conditioned by short-chain fatty acids: acetate, butyrate and propionate to further support IL-10 producing cells (107) or IgA production in B cells (108) (**Figure 2**).

Mucosal tolerance is maintained by Tregs activated in response to Myd88-dependent microbial sensing which promote IgA production in mouse models and enforce commensal bacteria (109). Myd88 regulates the expression of antimicrobial protein RegIIIγ preventing the activation of the adaptive immune response to bacteria (110).

Nod-like receptors such as NOD2 participate in shaping the microbiota composition by limiting the proliferation of the certain bacterial taxa in NOD2^-^/^-^ mice (111). In return, NOD2 can be stimulated by commensal bacteria to promote the survival of murine epithelial stem cells and epithelial renewal (112).

Commensal microbiota interacts with other innate immune effector cells like monocytes and macrophages to maintain homeostasis in the gut. Helicobacter hepaticus, a pathobiont, produces an immunomodulatory polysaccharide that induces IL-10 production in murine intestinal macrophages (113) and may further promote IL-10 producing Tregs (114)(**Figure 2**). Clostridium butyricum prevents acute experimental colitis in mice by inducing IL-10 production in intestinal macrophages (115). Anti-inflammatory response due to M2 polarization of macrophages is induced by Bacteroides fragilis (116) and Clostridia class, while Enterococcus faecalis induces a pro-inflammatory M1 phenotype (117, 118) (**Figure 2**).

Signals from microbiota can shape the phenotypic diversity and function of innate lymphoid cells ILCs. For example, microbial metabolite sensor Ffar2 regulates the proliferation and function of mouse type 3 ILCs (119). A study done by Kedmi et al. (120) showed that RORγt type 3 ILCs are necessary in inducing Tregs by the Helicobacter hepaticus showing that microbiota target multiple antigen presenting cells. Type 3 ILCs are important as immune surveillance players of the microbiota, participating in controlling the colonization resistance to pathogens by production of IL22 (121).

Although the role of the Th17 subset is controversial in the context of intestinal inflammation (122), their inflammatory activity may be determined by specific bacterial taxa inducing their differentiation. Segmented filamentous bacteria (SFB) induce non-inflammatory Th17 while Citrobacter rodentium induced production of pro-inflammatory cytokines from Th17 (123). Xu et al. demonstrated that TGF-β signaling drives the production of anti-inflammatory cytokine IL-10 in Th17 cells in the ileum and Peyers patches of the mice showing that plasticity of Th17 cells depends on environmental cues (124).

Another subset of T cells, follicular helper T cells (Tfh), can also play a role in microbiota composition. One study showed that deficiency of PD-1 co-receptor determines an altered phenotype of murine Tfh that affects IgA binding capacity to bacteria and results in an increase of the Enterobacteriaceae family (125). Tfh cells are crucial for GC formation in both mice and humans, assisting B cells in the generation of high affinity antibody production (126). Consequently, impairment in Tfh cells caused by lack of receptors, for example ATP-gated ionotropic P2RX7, translates to low IgA binding to commensals (127). On the other hand, microbiota can also have an impact on Tfh cells. Germ free mice have impairment in Tfh development, due to the loss of MYD88 signaling through TLR2 (128). Tfh cells are continuously exposed to microbial byproducts that infer in the GC reaction and the IgA production, participating in the regulation of the microbiota-host interaction (129, 130).

## Microbiota-T cells-IgA crosstalk in inflammation processes

Alterations in the gut microbiota due to multiple factors, such as environment or genetic susceptibility, contribute to development of intestinal inflammation and IBD. Multiple IBD loci have been linked to sensing and defense against bacteria, supporting the evidence of aberrant immune responses to intestinal microbiota (131–133). One of the first line of defense between luminal antigens and intestinal epithelium is represented by IgA production at mucosal surfaces (134). Although commensal bacteria induce homeostatic IgA production which in turn binds to bacterial communities to regulate their abundance and diversity (43, 135), higher levels of IgA were found on pathogenic and colitogenic bacteria (4).

In patients with IBD, bacteria taxa coated with IgA were shown to participate in the pathogenesis of inflammatory processes in mouse models (4, 6). Nevertheless, IgA coating plays a role in restricting pathogenic strains from disrupting the mucosal barrier (136, 137). Moreover, IgA coated bacteria from healthy individuals have immunosuppressive roles in the gut in comparison with taxa identified from IBD patients or from mice with dysbiosis. The outcome on intestinal inflammation from the two situations with IgA coated strains may be caused by different mechanism of T cell dependent IgA production. The inflammatory pathway is induced by Th17 cells while the immunoregulatory pathway depends on regulatory T cells (138).

Cong et al. (139) focused specifically on T dependent IgA production by Foxp3+Tregs in CBir1 Tg flagellin transgenic mice. The study showed that Foxp3+ Tregs when stimulated by a specific antigen only, in this case flagellin, promote the production of IgA in B cells. Upon depletion of Foxp3+ Tregs, IgA levels and IgA+ B cells were reduced in the lamina propria suggesting that Foxp3+ Tregs may also be important for the survival of intestinal IgA+ B cells. Foxp3+ T cells suppress inflammation and sustain a balanced and diverse microbiota, which in turn, stimulates the proliferation of Foxp3+ T cells and IgA production in a feedback regulatory loop (140). Foxp3+ T cells differentiate to T follicular regulatory (Tfr) cells and induce IgA production in B cells. When Tfr are absent, T follicular helper cells increase and affect the response of GC reaction towards more poly or self-reactive B cells (141, 142) (**Figure 1**). Furthermore, the production of a low specific IgA may determine changes in the microbiota and their microbial products that are necessary for the induction of regulatory T cells (143, 144). Th17 cells maintain homeostasis of the intestinal barrier in the steady state but can also act as a two-edged sword by promoting pathogenic inflammation in a genetic susceptible host (145, 146). IL-10 deficient mice have severe inflammation in the gut due to inflammatory and destructive processes mediated by Th17 responses (6). The study done by Britton et al. (138) offers a hypothesis regarding the immunoregulatory capability of microbiota in the etiology of IBD: the anti- or pro-inflammatory arm of the balance is influenced by the microbial communities in the gut which can either enrich a tolerogenic RORγt+Tregs cells or enrich proinflammatory Th17 cells (**Figure 1**).

IgA-seq helped to identify bacterial taxa coated with high levels of IgA from the gut of IBD patients (147) which were shown to worsen the development of DSS-induced colitis in gnotobiotic mice (4). However, the highly IgA coated bacteria are part of commensals and may be insufficient to drive the inflammation alone without having a specific susceptible environment or genetic conditions. For example, Prevotellaceae, one of the taxa identified by Palm et al. (4) aggravated chemically induced colitis in mice (148) and absence of IL-10 predisposed to colitis in the presence of Helicobacter species (149, 150).

## Regulatory B cells

B cells are involved in the generation of an effective immune response through antigen presentation, antibody and cytokine production. Interestingly, B cells can also become Bregs, which play a role in the suppression of the inflammatory milieu. The suppressive capacity has mainly been described by the production of IL-10. However, other immunoregulatory mechanisms are also employed (151, 152).

The phenotypic characterization of Bregs is challenging because of high heterogeneity in markers and lack of prototypic transcription factors which precisely identify the population. Consequently, Bregs are defined based upon their immunoregulatory function. B cells can become Bregs at different stages of development or differentiation, suggesting that the regulatory program may depend on environmental conditions and not on predisposed fate-decisions (153).

B cells involved in the first line of defense are represented by marginal zone B cells and B1 cells. To become mature, B cells have to travel from the bone marrow to the secondary lymphoid organs. As they progress to their maturation they go through 3 stages identified, in mice, as transitional B cells T1, T2 and T3. Transitional 2-marginal zone precursors (T2-MZP) were identified to produce IL-10 and have regulatory function *in vitro* and *in vivo* (154–156). Additionally, marginal zone CD23-CD1d^high^ B cells produce high amount of IL-10 and have suppressive activity (157–159). B10 cells are another population of Bregs, defined as CD1d^hi^ CD5+, and can be found in the spleen or peritoneal cavity (160–162). B10 produce solely IL-10 and originate either in the bone marrow or fetal liver (163). Antibody secreting cells (ASC), such as plasmablasts and plasma cells, can also become Bregs with important suppressive functions in experimental autoimmune encephalomyelitis (EAE) and a Salmonella infection model (164, 165). Other Breg subsets were described in mouse and humans with overlapping markers and suppressive function (166).

Bregs differentiate in response to inflammatory signals by expressing inhibitory markers and anti-inflammatory cytokines (167). Pro-inflammatory cytokines such as IL-6 or IL-1β are induced during inflammation and drive the differentiation of T2-MZP IL-10+ Bregs cells in a model of antigen induced arthritis. Interestingly, the production of IL-6 and IL-1β is regulated by the microbiota of mice manifesting arthritis (168). Nevertheless, IL10+ B cells can co-express IL10 together with pro-inflammatory cytokines including IL-6 and TNF-α, which may suggest a possible role of IL-6 and TNF-α for enhancing IL-10 production (169).

Factors such as B-cell activating factor (BAFF) and A proliferation-inducing ligand (APRIL) are involved in IgA B cell induction and survival (128, 170, 171), as well as in the induction of IL-10+ and IL-35+ Bregs during inflammation (172–176). Moreover, it was shown that human CD40^+^ILC3s engage in a feedback loop with B cells in which they induce the differentiation of Bregs through BAFF production (177) (**Figure 1**). The cytokine IL-21 is produced by Th17 and Tfh cells. Together with TGF-β or retinoic acid, IL-21 strongly promotes IgA class switching and production in mice (178). Furthermore, IL-21, IL-15 and granulocyte macrophage colony-stimulating factor (GM-CSF) are essential for the induction of Bregs (179, 180).

Although inflammatory stimuli induce the activation of Bregs, BCR recognition plays a critical role as well. By deleting two calcium sensors STIM1 and STIM2 in mice, causing a BCR impairment, Matsumoto et al. have shown that B cells produce less IL-10 due to defective BCR stimulation (181). IL-10 production by IL10+ plasmablasts requires engaging of TLR and BCR which further determine the transcription of IRF4 that binds to the IL-10 genomic locus (165). Another study found that the differentiation of B10 cells and IL-10 production can be induced by stimulating the B cells with LPS, PMA (Phorbol-12-myristat-13-acetat) and ionomycin (182). Moreover, Bregs are dominant in lymphoid tissues and the peritoneal cavity but their role can be hindered depending on the murine model of choice due to the presence of myeloid cells producing IL-10 in liver and blood. While IL10+ B cells play no essential role in endotoxemia, IL10+ B cells decrease the numbers of CD8+ T cells during infection with murine cytomegalovirus and control the immune activation when the mice were challenged with anti-IgD antibodies (183).

Bregs differentiation and IL-10 production depends also on CD40 stimulation and signaling and can be enhanced by IL-21 (169, 184). Follicular T helper cells express both IL-21 and CD40L in the GC, driving the class-switching of B cells to become memory B cells and plasma cells. Hence, the GC reactions might be an environment where effector and suppressive memory and plasma cells are produced (153).

An efficient immune response is comprised also of effective antigen presentation by B cells, which can further induce pro-inflammatory or anti-inflammatory reaction. For example, the lack of co-stimulatory ligands such as CD80 and CD86 on B cells induce T cell anergy or Tregs (153, 185). In return, Tregs can deplete CD80 and CD86 ligands from antigen presenting cells such as B cells and dendritic cells to further suppress the activation of conventional T cells (186).

Stimulation of B cells with IL-35, an anti-inflammatory cytokine, stimulates the conversion of B cells to IL35+ Bregs which further act on suppression of T cells (187). It was shown that in a model of experimental autoimmune uveitis both IL-10 and IL-35 signaling might be essential for the suppression function of IL35+Bregs (188). The same research group discovered that the IL-12p35 subunit has immune-regulatory functions and is responsible for inducing Bregs and Tregs expansion (189).

Several remaining questions still yet have to be answered regarding Bregs activation and differentiation. Is the fate of a Bregs written in their ontogeny or there are several factors that contribute to the conversion of B cells to Breg? Given the missing link of a transcription factor or the high diversity of markers that describe Bregs, is their state a stable one or it is transitory to a pro-inflammatory B cell? Moreover, looking at similar inducible cues for both Bregs and effector B cells, does it depend on a certain concentration or combination of factors? (153).

New studies addressing intracellular and extracellular metabolic signals in Breg offered some light in understanding the immunoregulatory balance of B cells. Although the microenvironmental signals seem to be similar for both regulatory and effector B cells, several pathways were identified as regulators of different outcomes. For example, gut and microbial metabolites (short-chain fatty acids, 5-hydroxyindoleacetic acid, fatty acids) particularly supportes regulatory B cell differentiation (166, 190, 191) (**Figure 2**).

## How does the microbiota interact with Bregs?

Microbiota promotes the differentiation of Bregs which in response suppress inflammatory signals. The regulatory function of Bregs has been described in several studies in autoimmune disease models of multiple sclerosis, rheumatoid arthritis and IBD (192–195). Bregs regulate the Th1/Th17 to Tregs balance in both mice and humans. Mice that have a conditional IL-10 deletion in B cells have reduced numbers of Tregs and an increase in pro-inflammatory Th1 and Th17 cells (156, 196) (**Figure 1**).

The suppressive function of Bregs is not only supported by their IL-10 production (166). Bregs can regulate the immune response through other mechanisms such as: TGF-β (197), EBI3/IL-35 (Epstein-Barr virus induced gene 3) participates in inducing Tregs (164, 189), GITRL (glucocorticoid-induced tumor necrosis factor receptor-related protein ligand) (194), FasL (Fas ligand) induced apoptosis by binding to its receptor (198), PD-L1 (programmed death ligand 1) participate in restricting T cell differentiation (173), CD73 has an immunosuppressive effect by converting adenosine monophosphate to adenosine (199, 200). B cells cooperate with Tregs in feedback loops to attenuate inflammatory processes in the gut. In DSS-colitis, B cell deficient mice have lower number of Tregs in GALT and display more severe colitis. Interestingly, B cells attenuated colitis in an IL10-independent pathway and induced the differentiation of Tregs which supported the functions of IgA-plasma cells (25).

The immunogenicity of bacteria seems to play an important role in B cell activation and differentiation during the interaction of B cells with host microbiota. If the encountered bacteria have a strong immunogenic potential then Bregs will respond with a strong immunoregulatory response to maintain immune tolerance (201). Although, a more intense suppressive response by Bregs can regulate pro-inflammatory immune responses caused by the same bacteria, in the absence of Bregs the strong immunogenic bacteria will aggravate the disease progression (202–204).

Defects in PI3Kγ signaling translate to reduced numbers of IL-10 producing Bregs in response to resident bacteria. B cells lacking PI3Kγ were unable to resolve the intestinal inflammation in T cell mediated-colitis (205). The human IBD7 susceptibility locus harbors the PIK3cd gene (206, 207) and genetic deletion of PI3Kγ drives spontaneous colitis in the presence of commensals (208, 209). These findings might explain the roles of specific pathways in the activation of immune cells by microbiota, since the PI3Kγ pathway seems to contribute to the immunoregulation mostly when intestinal microbiota is the main activator of inflammation (205). Another study done by Mishima et al. supports the role of PI3Kp110d signaling for IL-10 production by bacteria murine activated-B cells and its importance in maintaining mucosal tolerance (210). They also observed that continuous bacterial stimulation is essential for mounting a sustained activation of Bregs *in vitro*.

Breg cells are characterized by their ability to suppress the immune system, but their high diversity in phenotypic marker and transcription factor expression makes specific phenotyping difficult (152). The transcription factor AhR (aryl-hydrocarbon receptor) contributes to the interaction of immune cells and microbiota by binding diverse ligands from dietary components or from microbiota (211). Recently, several studies revealed that Ahr drives and maintains the immunoregulatory function of splenic Breg (212, 213). Importantly, Piper et al. shows that, in mice, Ahr responds to inflammatory stimuli and promotes the differentiation of Bregs by regulating the production of IL-10 and silencing the transcription of pro-inflammatory cytokines (212). Moreover, Rosser et al. shows that presence of gut microbiota derived metabolites such as butyrate increases the Ahr ligand availability which results in supporting Breg function (190).

In addition to IL-10; other cytokines such as IL-35 are essential in the outcome of several diseases (194). IL35+ B cells interact with microbiota through their microbial metabolites and maintain intestinal homeostasis. A microbial metabolite, indoleacetic acid, supports the expansion of IL35+ Bregs which are the main source of IL-35 in the intestine during DSS-induced colitis. In turn, the presence of IL-35 has immunosuppressive function and shapes the intestinal microbiota (214). Moreover, IL-35 production plays an important role in reducing the inflammation in models of T cell dependent colitis (215) and DSS-induced colitis (216). Although the IL-35 secretion is decreased in patients with UC compared to controls, higher IL-35 in the mucosa of UC was correlated with higher IL-10 secretion in the gut (217). Moreover, B cells from CD patients have a higher expression of IL-35 but lower protein secretion compared to controls (218).

Several bacterial pathogens can induce immunosuppressive response to infection as an immunological escape. For example, Helicobacter pylori infection can induce differentiation of Foxp3+ Tregs and CD19+ IL10+Bregs (219). When mice are infected with H. pylori and treated with DSS-colitis, the colitis scores were significantly reduced with less pro-inflammatory cytokines in the colonic mucosa (220, 221). The studies suggest the role of H. pylori in attenuation of acute and chronic colitis. Salmonella sp. infection helped to identify a subset of IL-35 plasma cells which is induced during infection together with IL-10 plasma cells and highlighted the role of IL-35 Bregs as essential regulators of the immune system (164, 222).

## IgA Bregs

Although IgA plasma cells modulate the immune response in particular at the mucosal sites through production of IgA, their role as immunosuppressive players is not yet fully understood.

*In vitro* studies showed that IL-10 stimulation of human but not mouse B cells contributes to proliferation of activated B cells and may drive IgA production (223, 224). Kunisawa et al. (225) found that IL-10 induced CD11b+, IgA+ plasma cells and determine IgA production on those cells. Although it is not clear if IL-10 alone is necessary for IgA production or some other factors are participating in maintaining CD11b+ IgA+ plasma cells, the study identified the CD150 surface marker as potential mechanism through which IL-10 can mediate B cell proliferation. CD11b+ B cells are present in the GALT in DSS-induced colitis and in UC patients. CD11b+ B cells play an immunosuppressive role by inhibiting colitis in mice and produce increased levels of IgA (226) (**Figure 2**).

As described before, another immunosuppressive mechanism of Bregs is through expression of PD-L1 upon sensing inflammatory signals (227, 228). IgA plasma cells in the lamina propria of mice were found to express PD-L1 and to induce FOXP3+ Tregs cells but not pro-inflammatory IFN-γ+ Th1 cells (229) (**Figure 2**). Despite the fact that IgA production is not the main focus, one study by Serrán et al. (230) found expression of high levels of PD-L1 on plasmablasts during infection with *Trypanosoma cruzi* with a suppressive effect on T cells.

Fehres et al. identified a new subset of human IL-10 producing B regs that express IgA (173). In this study, they describe that APRIL stimulation determines differentiation to IgA+ B regs, while other stimulations such as TGF-β and BAFF does not induce IL-10 production in IgA+ Bregs. APRIL-induced IgA+ Bregs significantly, suppressed CD4+ proliferation and induced Foxp3+ Tregs. Furthermore, APRIL-induced IgA+ Bregs also inhibited TNF production by macrophages through IL-10 and PD-L1. Albeit this role of APRIL for production of IL-10 by IgA+ Bregs, some other studies identified TGF-β as potent stimulatory factor for differentiation of immunosuppressive plasma cells which express IgA, IL-10 and PD-L1 in a mouse model of prostate cancer (231).

Non-resolving inflammatory responses in IBD are associated with the development of colitis-associated colorectal cancer (CAC) (232, 233). Even though IL-10 deficiency in B cells aggravates the outcome of DSS-induced chronic colitis, it does not contribute to the development of tumorigenesis in CAC, which suggests the presence of other immunosuppressive mechanisms. Melcher et al. found that Bregs can exhibit dual protective phenotypes: IL-10 production for the suppression on inflammatory environment and on IgA production for shaping a protective microbiota (234)(**Figure 2**). Interestingly, in this study the IL-10 producing Bregs exert suppressive effects on Th1/Th17 cells in mouse chronic colitis and then differentiate into IgA+ plasma cells in response to TLR activation. After the differentiation into IgA+ plasma cells, the Bregs lost the capacity for IL-10 production.

Homeostasis in the colon may also depend on the interaction of IgA antibody secreting cells (IgA-ASC) and Tregs. IgA-ASC that express the gut homing chemokine receptor CCR10+ form conjugates with Tregs to direct their migration into the colon to support a homeostatic environment (235). Moreover, CCR10+ IgG1-ASC have immunoregulatory role and can compensate for the loss of CCR10+ IgA-ASC in IgA-ko mice. Furthermore, IgA-ASC originate in the gut in response to commensals and can migrate to central nervous system during experimental autoimmune encephalomyelitis (EAE) where they play a critical role in regulation of inflammation (236).

Bregs have heterogeneity in phenotypic markers and express different transcription factors in both mice and human, giving rise to the hypothesis that Bregs do not comprise a distinct cellular lineage but any B cells can differentiate to Breg cells in an appropriate environment (167). Latest research show that IgA production by Bregs may be a new mechanism to regulate the immune response and may bring new light in understanding the anti-inflammatory and pro-inflammatory balance.

## Pro-inflammatory IgA and IgG

IBD patients have increased numbers of B cells in the inflamed intestine compared to healthy individuals. Interestingly, UC patients described as ulcerative colitis type 1, have increased expression of the B cell activation factor BAFF, suggesting a possible role for B cells in IBD. Further studies have also confirmed the expansion of B cells in UC patients. In a study done by Rubin et al. they observed more gut trophic CD45RO+ B cells in the inflamed tissue of CD patients compared to UC patients (237). These CD45+RO B cells are considered a biomarker for CD activity index and permeability of the gut. Moreover, a lower expression of pSTAT3 levels in CD25+ B cells in inflamed tissue of CD patients was detected compared to uninflamed tissue which can be associated with lower levels of regulatory B cells (237). The study of Pararasa et al. suggests that the increase in CD27-IgD- memory B cells in GALT of IBD patients is rather determined by the proliferation at the site of inflammation and less by the recruitment of cells from the blood. However, is interesting to notice the same immune response for both UC and CD, supporting the notion that the response to intestinal challenge determine the expansion of CD27- B cells (238). The inflamed gut of UC patients has increased IgA+ and IgG+ plasma cell populations compared with healthy controls. Uzzan et al. suggests that IgG+ plasma cells could participate in enhancing the inflammatory milieu (8). The increased number of B cells during chronic inflammation in the intestine develops in lymphoid structures recently described as tertiary lymphoid structures (TLSs). However, TLS are present more in biopsies from CD patients and still remain enigmatic in their role during the disease (239, 240).

IgA produced at mucosal sites is considered to have anti-inflammatory proprieties by keeping tolerance to commensals and protecting against pathogens. Despite its high abundance in the blood, the role of IgA in the serum is less understood (241). In recent years the role of IgA binding to FcαRI (CD89) gained increasing attention. FcαRI expression is observed in neutrophils, eosinophils, macrophages, monocytes and Kuppfer cells (242). Hansen et al. show that serum IgA forms immune complexes, binds to FcαRI and induces pro-inflammatory cytokine production in different human myeloid cells (243). High levels of IgA immune complexes, IgA autoantibodies or increased IgA levels in the serum are found in diseases such as celiac disease (244), IgA nephropathy (245); and IBD (246).

Furthermore, patients with IBD have increased levels of commensal-targeting IgG in the serum which contributes to inflammation of the intestinal mucosa (247). Uzzan et al. found reduced VDJ gene mutation in IgG+ plasma cells in UC which might suggest an impairment of GC for differentiation of IgG+ and IgA+ plasma cells due to hyperstimulation of B cells in an inflammatory environment or might be explained by higher accumulation of autoreactive cells due to inflammation (8). A variant of FCGR2A, which alters the binding affinity of the antibody receptor it encodes, FcγRIIA, for IgG, has been found in genome-wide association studies (GWAS) in UC. The study of Castro-Dopico et al. shows that colonic mucosa of UC patients with FCGR2A genotype have anti-commensal IgG which forms complexes with macrophages and induces NLRP3 and reactive oxygen species with the production of pro-inflammatory IL-1β and neutrophil-recruiting chemokines (248).

## Conclusion

In conclusion, the cytokine network in intestinal inflammation leading to IBD is complex and highly regulated by genetic, microbial, and immune factors. So far therapeutic agents targeting cytokines have at least in some patients limited efficacy and varied outcomes. Targeting all B cells with Rituximab has proven to be unsuccessful and rather detrimental to IBD patients, most likely particularly because potential functions of Bregs have been overlooked (12). Better understanding the proinflammatory role of B cells and the factors that induce intestinal Bregs could help to design better treatments that do not interfere with the activity of beneficial Bregs and provide new insights into stimulating Bregs to tilt the balance towards suppression of inflammation. The emergence of IgA-producing Bregs adds another mechanism for regulating inflammation in close interaction with the microbiota. Further research is needed to fully comprehend the intricate mechanisms and interactions within this complex network and to develop more effective treatments for IBD.

## Author contributions

RZ: Writing – original draft. SW: Writing – review & editing.
